# Rethinking Electronic Effects in Photochemical Hydrogen Evolution Using CuInS_2_@ZnS Quantum Dots Sensitizers

**DOI:** 10.3390/molecules27238277

**Published:** 2022-11-27

**Authors:** Antonio Orlando, Fiorella Lucarini, Elisabetta Benazzi, Federico Droghetti, Albert Ruggi, Mirco Natali

**Affiliations:** 1 Department of Chemical, Pharmaceutical and Agricultural Sciences (DOCPAS), University of Ferrara, Via L. Borsari 46, 44121 Ferrara, Italy; 2 Department of Chemistry, University of Fribourg, Chemin de Musée 9, CH-1700 Fribourg, Switzerland

**Keywords:** hydrogen production, cobalt polypyridine catalysts, artificial photosynthesis

## Abstract

Molecular catalysts based on coordination complexes for the generation of hydrogen via photochemical water splitting exhibit a large versatility and tunability of the catalytic properties through chemical functionalization. In the present work, we report on light-driven hydrogen production in an aqueous solution using a series of cobalt polypyridine complexes as hydrogen evolving catalysts (HECs) in combination with CuInS_2_@ZnS quantum dots (QDs) as sensitizers, and ascorbate as the electron donor. A peculiar trend in activity has been observed depending on the substituents present on the polypyridine ligand. This trend markedly differs from that previously recorded using [Ru(bpy)_3_]^2+^ (where bpy = 2,2’-bipyridine) as the sensitizer and can be ascribed to different kinetically limiting pathways in the photochemical reaction (*viz*. protonation kinetics with the ruthenium chromophore, catalyst activation via electron transfer from the QDs in the present system). Hence, this work shows how the electronic effects on light-triggered molecular catalysis are not exclusive features of the catalyst unit but depend on the whole photochemical system.

## 1. Introduction

Hydrogen production via light-driven water splitting is recognized as a candidate fundamental reaction for the generation of clean fuels [1,2]. For this purpose, one of the simplest approaches envisions the coupling in an aqueous solution of three molecular components, namely a light-harvesting sensitizer, a hydrogen evolving catalyst (HEC), and an electron donor [3,4,5]. Ideally, this latter component should be constituted by the water molecule itself, leading to the production of oxygen. However, the intrinsic kinetic hurdles associated with the 4-electron, 4-proton management of the oxygen-evolving reaction (OER) renders the anodic half reaction too slow for applicative purposes. A sacrificial electron donor, *viz*. a molecule undergoing decomposition upon one-electron oxidation [6], is usually employed to enable the light-driven hydrogen evolving reaction (HER) to proceed at sustained rates.

Within the aforementioned three-component system, the reaction scheme usually requires a sequence of events involving (i) the excitation of the sensitizer, (ii) reductive quenching of the excited state by the sacrificial electron donor, (iii) activation of the HEC via two step-wise electron transfer processes from the reduced sensitizer [5]. Common light-harvesting units employed for the light-driven HER are molecular sensitizers such as coordination compounds, e.g., [Ru(bpy)_3_]^2+^ (where bpy = 2,2′-bipyridine), [7,8,9,10,11], copper(I) or iridium(III) complexes [12,13], or metalloporphyrins [4,14]. However, the low stability under prolonged irradiation typically limits the achievement of sufficiently large turnover numbers (TONs). Starting from these premises, within the last few years much attention has been paid to the preparation of quantum dots (QDs), *viz.* semiconductor nanocrystals stabilized by suitable capping agents, for replacing molecular sensitizers since they combine several key properties such as controllable optical properties, high emission yield, sufficiently long excited state lifetime, and improved stability under redox stress thanks to their material-based nature [15,16]. In this regard, many studies have preferentially dealt with the use of cadmium chalcogenides due to their synthetic ease and tunability [17,18,19,20,21]. However, the toxicity of cadmium strongly prevents application in the green chemistry context. In order to tackle this issue, only recently have cadmium-free QDs been successfully applied in energy conversion schemes [22,23,24,25]. Of remarkable interest, core-shell CuInS_2_@ZnS nanocrystals proved to be extremely active and durable for the production of hydrogen under irradiation in the presence of a molecular cobalt tetrazamacrocyclic complex as the HEC and ascorbate as the sacrificial electron donor [26].

**Figure 1:**
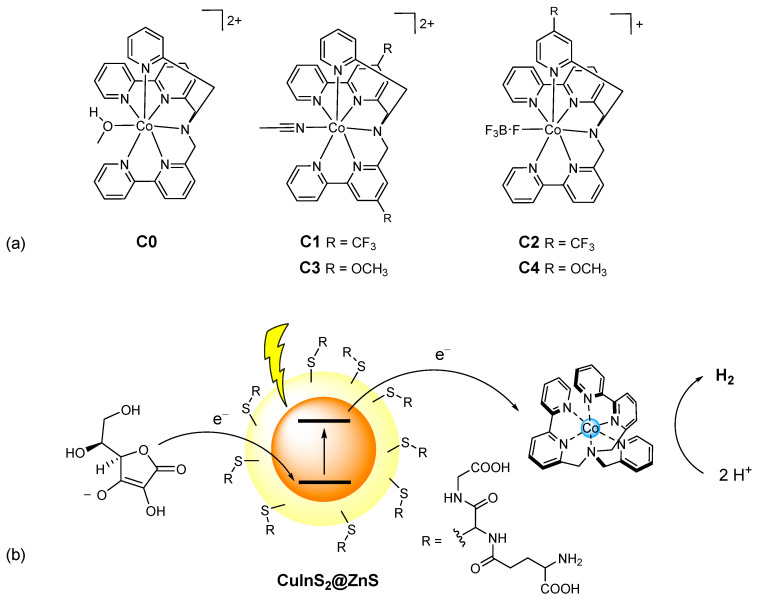
(**a**) Molecular structure of the cobalt complexes **C0–4**; (**b**) schematic representation of the photochemical system here examined involving glutathione-capped core-shell CuInS_2_@ZnS QDs as the sensitizer, ascorbate as the sacrificial donor and the cobalt complexes **C0–4** as the HECs.

The stability of a photochemical system for hydrogen evolution requires specific care on the catalytic routine as well. Within this framework, cobalt polypyridine complexes have demonstrated improved stability over molecular analogues under operation in aqueous conditions [5]. We have recently reported on a series of cobalt polypyridine complexes **C0–4** featuring hexadentate ligands with diverse electronic substituents on either the bipyridine or the pyridine groups (Figure 1a) [27]. 

These complexes were tested as HECs under photochemical conditions using [Ru(bpy)_3_]^2+^ as the sensitizer and ascorbate as the sacrificial agent leading to remarkable TONs up to 5520 with turnover frequencies (TOFs) of up to 86.1 min^−1^, and a quantum yield of 11.3%. Interestingly, a nice relationship between the position of the substituent and the photosynthetic activity by the corresponding three-component system was found (**C4 > C2 > C0 > C3 > C1**), which was mainly interpreted with the aid of DFT computations, on the basis of the position of the substituents, which plays a key role on the kinetics of the protonation steps required in the ECEC catalytic mechanism (where E and C are reduction and protonation steps, respectively) by such cobalt complexes.

In the present work, we report on light-driven hydrogen evolution promoted by the same series of cobalt complexes **C0–4** in an aqueous solution using glutathione-capped core-shell CuInS_2_@ZnS QDs as the sensitizer and ascorbate as the sacrificial donor. Interestingly, the photosynthetic activity by this class of complexes displays a particular trend that reflects the electronic nature of the substituents and markedly differs from that previously recorded using [Ru(bpy)_3_]^2+^ as the sensitizer. This is associated with different limiting kinetic regimes with the two different photosensitizers employed and highlights how the electronic effects on light-triggered molecular catalysis depend on the entire characteristics of the photochemical system.

## 2. Results

**Figure 2:**
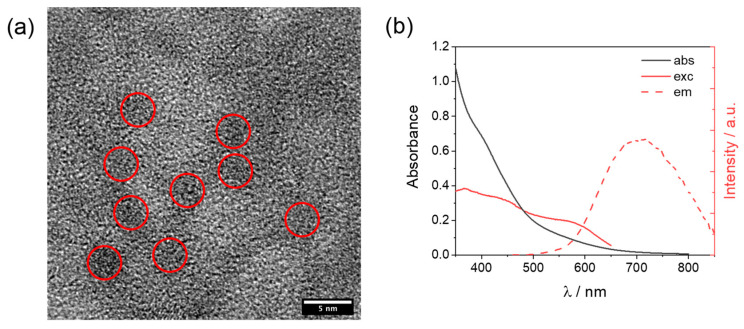
(**a**) High-resolution TEM image of aqueous CuInS_2_@ZnS QDs with red circles identifying discrete nanoparticles; (**b**) absorption, excitation (recorded emission at 700 nm), and emission (excitation at 450 nm) spectra of CuInS_2_@ZnS QDs in water.

The cobalt complexes were available from the previous study and were prepared by treatment of the hexadentate ligand **L0–4** with Co(BF_4_)_2_·6H_2_O in methanol, followed by precipitation with diethyl ether. Details of the synthetic protocols for both ligands and complexes, as well as for the corresponding characterization, can be found in the original reference [27]. The glutathione-capped core-shell CuInS_2_@ZnS QDs were prepared using microwave heating, following the same literature protocol reported by Collomb and co-workers [26]. The procedure (see Materials and Method for details) involves the construction of the CuInS_2_ core as a first step, followed by the formation of the ZnS shell as a second step, and the final precipitation of the core-shell CuInS_2_@ZnS QDs. The as-prepared QDs were then characterized by transmission electron microscopy (TEM) and optical spectroscopy. High-resolution TEM images (Figure S1) show that the nanocrystals are highly aggregated, probably being embedded in an organic amorphous matrix derived from the glutathione ligand framework. When the size resolution is increased up to 5 nm some darker regions, associated with discrete nanoparticles (red areas, Figure 2a), can be distinguished, featuring an average diameter of about 3 nm. This attribution is indeed confirmed by the observation of crystalline features during image acquisition (Figure S2), whose diffraction patterns display a 3.1 Å d-spacing characteristic of chalcopyrite phases [28,29]. EDS analysis (Figure S3) exhibits the emission patterns of Cu, In, S, and Zn, supporting the expected elemental composition of the QDs. The absorption spectrum in an aqueous solution (Figure 2b) shows a detectable absorption starting at about 700 nm, which progressively increases in absorbance with the shortening of the wavelengths. No clear bands can be discerned, as expected based on the absence of clear excitonic transitions for CuInS_2_ [30,31]. The as-prepared QDs display red luminescence with an emission maximum of 710 nm (Figure 2b). A quantum yield of ~1% can be calculated using [Ru(bpy)_3_]^2+^ in water as a standard (Φ = 2.8%). Interestingly, the excitation spectrum (Figure 2b) exhibits a clear band with a maximum of 577 nm and suggests that the emission mostly arises from the excitation of the absorption edge above 500 nm. These data are fully compatible with those expected for the formation of core-shell CuInS_2_@ZnS QDs [26]. The red shift of the emission band and the slight decrease in the quantum yield with respect to what was reported in the original reference [26] very likely reflect the obtainment of larger CuInS_2_@ZnS QDs in our case (~2 nm vs. ~3 nm diameter for the literature and the present sample, respectively). Time-resolved luminescence measurements (excitation at 532 nm) were conducted in order to evaluate the excited state decay of our CuInS_2_@ZnS QDs (Figure S4). The decays were measured at three different wavelengths (650, 700, and 740 nm) and satisfactorily fitted using a triexponential function [26]. Amplitude-weighted average lifetimes of 85, 105, and 120 ns were calculated at 650, 700, and 740 nm, respectively, showing a slight increase when moving toward the red portion of the visible spectrum. Both the multiexponential fitting and the variable average lifetimes recorded at the three different wavelengths are consistent with some size polydispersion, as well as the presence of different intraband trap states, as typically observed for this kind of materials [32,33]. The emission band can indeed be reasonably reproduced using two Gaussian functions (Figure S5) associated with the emission from two different electronic states. Most importantly, the emission decays are sufficiently long lived to efficiently let the present CuInS_2_@ZnS QDs partake in the bimolecular reactions required for light-driven hydrogen evolution under homogenous conditions in combination with an HEC and a sacrificial donor.

Light-driven hydrogen evolution experiments were conducted upon visible-light irradiation (400–800 nm) of a 5 mL aqueous solution containing CuInS_2_@ZnS QDs as the sensitizer, the cobalt complexes **C0–4** as the HEC, and ascorbate as the sacrificial electron donor. A fixed concentration of both QDs (0.11 mM) and ascorbate (0.5 M) was used in all photochemical tests. The experimental data obtained in terms of moles of hydrogen produced as a function of the irradiation time (kinetic traces) were properly employed in order to extract the relevant photocatalytic parameters [5]: (i) the maximum turnover number (TON) calculated as the ratio between the maximum quantity of hydrogen produced (in moles) and the quantity of catalyst employed (in moles), (ii) the maximum turnover frequency (TOF/h^−1^) calculated as the ratio between the initial rate of hydrogen production (in mol/h, estimated in the linear portion of the kinetic trace) and the moles of catalyst, and (iii) the quantum efficiency (QE/%) calculated as the ratio between two-times the initial rate of hydrogen formation (in mol/h) and the absorbed photon flux (in Einstein/h).

**Figure 3:**
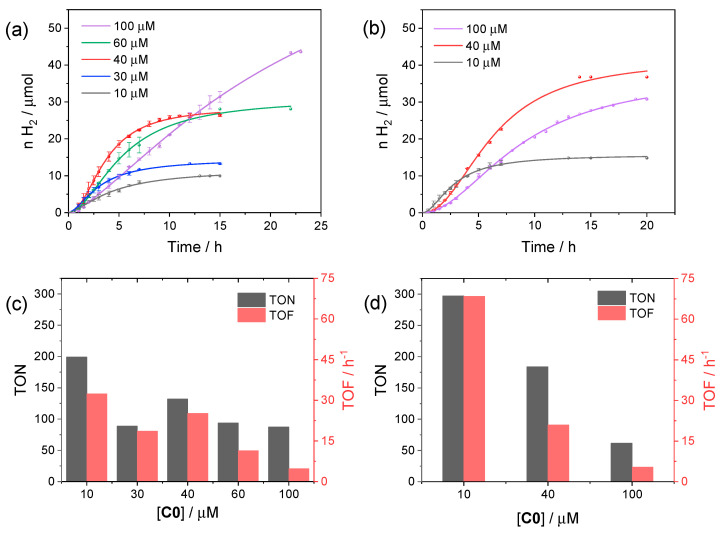
Kinetics of hydrogen evolution obtained upon visible light irradiation of aqueous solutions (5 mL) containing 0.11 mM CuInS_2_@ZnS QDs, 0.5 M ascorbate, and 10–100 µM **C0** at (**a**) pH 4 and (**b**) pH 5; photocatalytic data at (**c**) pH 4 and (**d**) pH 5 in terms of maximum TONs after 24 h and TOFs.

We first performed photocatalytic tests using **C0** as the reference catalyst in order to find the optimized conditions (catalyst concentration and pH) to profitably compare the resulting catalytic activity within the series of substituted cobalt complexes. In this respect, we started to examine the photochemical activity at pH 4, *viz*. the optimum pH identified in the three-component system involving [Ru(bpy)_3_]^2+^ as the sensitizer [34,35], and varied the concentration of **C0** from 10 to 100 µM. The corresponding kinetic traces are reported in Figure 3a, while the relevant photocatalytic data are depicted in Figure 3c.

From the comparison of the kinetic traces (Figure 3a), both the amount of hydrogen produced at plateau and the rate of hydrogen production increase when moving from 10 to 40 µM **C0** concentration. Then, the rate decreases when moving from 40 to 100 μM, likely as a result of short-circuiting events induced by the large catalyst concentration, although a larger amount of hydrogen can be produced with a longer irradiation time at 100 µM **C0**. The photocatalytic data, in terms of TON and TOF (Figure 3c), show the largest values for small concentrations of catalyst, as typically observed for analogue photochemical systems [36], reaching maximum values of 200 and 33 h^−1^, respectively, at 10 µM. Interestingly, similar results were obtained at pH 5 (Figure 3b), under which conditions both improved TONs and TOFs were achieved (maximum values of 298 and 72 h^−1^, respectively, at 10 µM catalyst concentration, Figure 3d).

We then selected the concentration of 40 µM **C0**, which represents the condition leading simultaneously to both a higher rate and amount of hydrogen, in order to examine the dependence of the photocatalytic performance on pH (Figure S6). As can be seen from the kinetic data, the photochemical system evaluated at pH 4 and 5 displays comparable results, with a slight improvement at higher pH, in terms of the amount of hydrogen produced at plateau, but at the expense of slightly lower rates (see also the full comparison at different catalyst concentration in Figure 3). On the other hand, an additional increase of pH up to 6 leads to a remarkable decrease in performance. Finally, precipitation of the CuInS_2_@ZnS QDs occurs below pH 4 due to protonation of the glutathione capping agent, a situation that prevents reliable tests at more acidic pH values.

**Figure 4:**
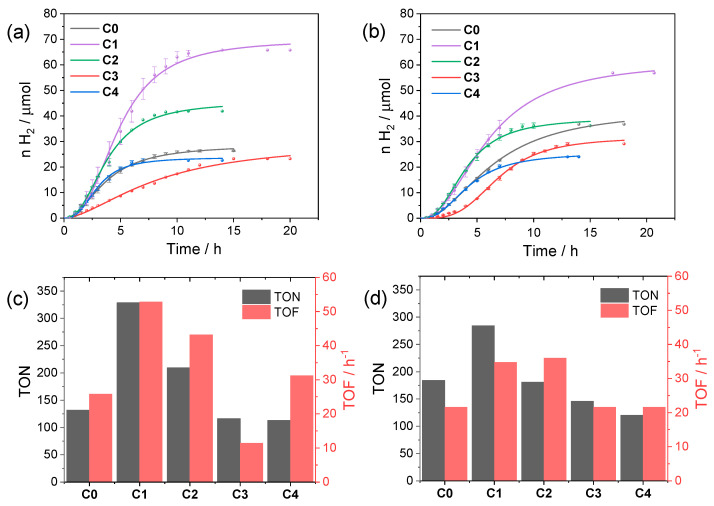
Kinetics of hydrogen evolution obtained upon visible light irradiation of aqueous solutions (5 mL) containing 0.11 mM CuInS_2_@ZnS QDs, 0.5 M ascorbate, and 40 µM **C0–4** at (**a**) pH 4 and (**b**) pH 5; photocatalytic data in terms of maximum TONs after 24 h and TOFs at (**c**) pH 4 and (**d**) pH 5.

According to these data, we selected both pH 4 and 5 and the concentration of a 40 µM catalyst as reference conditions to evaluate the performances of the **C0–4** series in light-driven hydrogen evolution using CuInS_2_@ZnS QDs. Figure 4 collects the kinetic traces and the resulting photocatalytic data obtained upon irradiation. The photochemical activity is markedly affected by the nature of the catalyst as expected based on the chemical difference and the position of the substituents. At pH 4 (Figure 4a,c) the most active catalyst is complex **C1** (maximum TON = 329, TOF = 53 h^−1^, QE = 0.29%), followed by complex **C2** (maximum TON = 210, TOF = 43 h^−1^, QE = 0.22%), both featuring –CF_3_ substituents. Complexes **C0** and **C4** perform similarly (maximum TON = 132 and 113, TOF = 26 and 31 h^−1^, QE = 0.14% and 0.16%, respectively), while **C3** displays the lowest performances in the series (maximum TON = 116, TOF = 11 h^−1^, QE = 0.06%).

The trend in activity is apparently confirmed at pH 5 (Figure 4b,d), although the differences are slightly attenuated with respect to pH 4. As a matter of fact, under these conditions, **C1** (maximum TON = 285, TOF = 35 h^−1^, QE = 0.20%) still performs better than the remaining complexes (maximum TON = 184, TOF = 22 h^−1^, QE = 0.12% for **C0**; maximum TON = 181, TOF = 36 h^−1^, QE = 0.20% for **C2**; maximum TON = 146, TOF = 22 h^−1^, QE = 0.12% for **C3**; and maximum TON = 120, TOF = 22 h^−1^, QE = 0.12% for **C4**).

**Figure 5:**
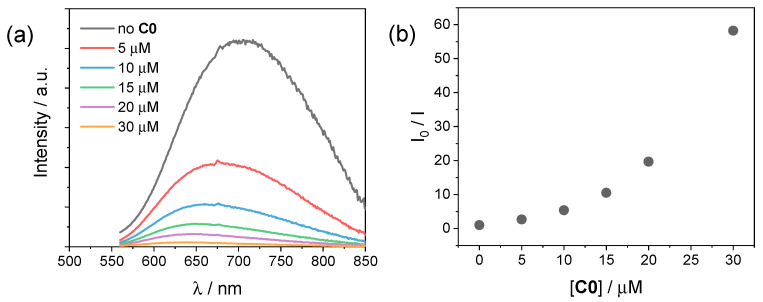
(**a**) Luminescence spectra (excitation at 450 nm) of CuInS2@ZnS QDs in water upon addition of 0–30 µM **C0**; (**b**) plot of the I_0_/I ratio vs. **C0** concentration.

In order to investigate the photochemical system employed for the hydrogen evolution experiments, we performed luminescence studies on our CuInS_2_@ZnS QDs in the presence of either the cobalt complexes **C0–4** or the ascorbate donor. Figure 5a depicts the emission spectra of CuInS_2_@ZnS QDs in an aqueous solution in the presence of different concentrations of **C0**. The addition of small quantities of cobalt complex brings about a decrease in the luminescence accompanied by an apparent blue shift of the emission maximum. The plot of the ratio between the emission intensity in the absence (I_0_) and in the presence (I) of the cobalt complex is not linear (Figure 5b). This observation can be possibly ascribed to (i) a strong contribution from a static quenching phenomenon due to interactions between the CuInS_2_@ZnS QDs and the cobalt complex, favored by the complementary charges of the two partners (negative for the QDs, due to the presence of the deprotonated glutathione capping agents; positive for the cobalt complex, Figure 1), (ii) the quenching of different electronic states of the CuInS_2_@ZnS QDs [32], consistent with the blue shift of the emission maximum, or, most likely, (iii) a combination of both factors. The observed quenching can be presumably associated with the occurrence of an electron transfer from the conduction band (CB) of the QDs to the cobalt complex, although alternative mechanisms such as energy transfer cannot be ruled out [37,38]. A similar behavior is exhibited by all the remaining cobalt complexes **C1–4** (Figures S7–S10), with only minor differences in quenching yield possibly associated with the different nature of the complexes and the resulting interactions with the QD surface. Besides suggesting profitable charge transfer events occurring upon excitation of CuInS_2_@ZnS QDs in the presence of the cobalt complexes, under these premises the luminescence data cannot be properly employed to infer the efficiency of the electron transfer process from the excited QDs to the cobalt catalyst, namely the first electron transfer event initiating light-driven HER catalysis.

The quenching of the luminescence of CuInS_2_@ZnS QDs by the ascorbate donor was then examined at pH 5. A linear plot was obtained by plotting the I_0_/I ratio vs. the ascorbate concentration (Figure S11), consistent with bimolecular electron transfer involving oxidation of the donor by the holes in the valence band (VB) of the QDs. Linear fitting of the experimental data according to the Stern-Volmer equation leads to a K_SV_ = 0.24 s^−1^. Considering an average lifetime of τ = ~100 ns (see above), a bimolecular rate constant of *k* = ~2.4·10^6^ M^−1^s^−1^ can be estimated for such an electron transfer process. This value is one order of magnitude lower than the bimolecular reductive quenching of [Ru(bpy)_3_]^2+^ by the same sacrificial donor [35]. This could be due to the presence of negative charges on the surface of the QDs, that repel the ascorbate anion thus lowering the chances of a diffusional encounter, as well as to thermodynamic reasons. At the ascorbate concentration employed in the hydrogen evolution experiments (0.5 M), this bimolecular rate constant translates into a ~10% efficiency of the ascorbate oxidation by CuInS_2_@ZnS QDs upon irradiation.

## 3. Discussion

The cobalt complexes **C0–4** were reported as active molecular catalysts for the generation of hydrogen from an aqueous solution under photochemical conditions using [Ru(bpy)_3_]^2+^ as the sensitizer and ascorbate as the sacrificial electron donor. TONs between 591–5520, TOFs between 26.7–86.1 min^−1^, and QEs between 3.5–11.3% were recorded depending on the catalyst used [27]. Improved performances were achieved using cobalt complexes **C2** and **C4** than **C1** and **C3** with the parent compound **C0** displaying intermediate activity. The observed differences were mainly associated with the position of the substituent and the resulting effect on the protonation kinetics with the corresponding electronic nature playing a less relevant role. According to an ECEC catalytic mechanism, DFT computations confirmed that the formation of the relevant Co(II)-H catalytic intermediate, occurring via internal proton transfer from a Co(0)LH species, featuring a formally doubly-reduced metal center and a protonated ligand, was more favorable for the pyridine-substituted complexes **C2** and **C4** with respect to the bipyridine-substituted analogues **C1** and **C3** [27].

In the present work, the activity of this class of molecular catalysts has been tested using CuInS_2_@ZnS QDs as sensitizers under similar experimental conditions. Overall, the performances in light-driven hydrogen production are lower than those measured using the molecular [Ru(bpy)_3_]^2+^ sensitizer, particularly as far as the rate of hydrogen evolution is concerned. TOFs values between 22–53 h^−1^ and QEs between 0.12–0.29% have been indeed recorded for the present photochemical systems, which are orders of magnitude lower than those measured for the ruthenium-based three-component systems. This experimental evidence can be reasonably attributed to both the slow kinetics for the ascorbate oxidation (bimolecular rate of *k* = ~2.4·10^6^ M^−1^s^−1^, corresponding to only ~10% efficiency at the ascorbate concentration used) and the lower reduction ability of the photogenerated reductant, namely the reduced [Ru(bpy)_3_]^+^ sensitizer in the previous system (E = −1.34 V vs. SCE) [39] and the photogenerated electrons in the CuInS_2_@ZnS QDs (E = −1.12 V vs. SCE for the conduction band edge) [26]. The above observations and considerations also suggest that the rate-determining step in the light-driven catalysis by the **C0–4** series can be potentially different depending on the sensitizer employed.

**Figure 6:**
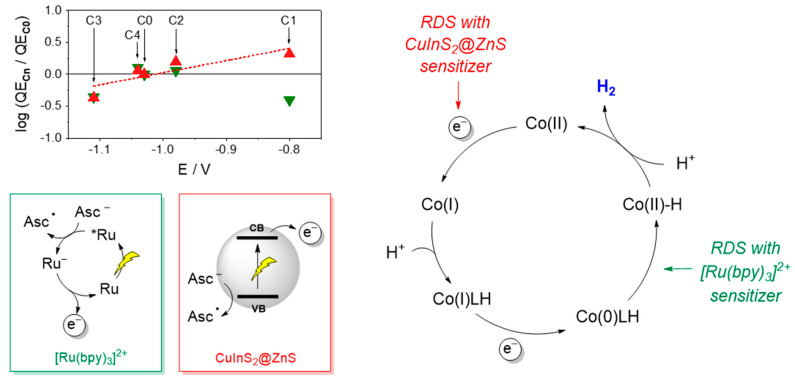
Plot of the log(QE**_Cn_**/QE**_C0_**) (where n = 0–4) at pH 4 vs. the half-wave potential of the catalytic wave in acetonitrile for **C0–C4** (see Ref. [27]) for the light-driven catalytic data obtained with the photochemical systems based on core-shell CuInS_2_@ZnS QDs (red triangles) and [Ru(bpy)_3_]^2+^ (green triangles) as the sensitizers and schematic representation of the main processes and rate-determining steps involved in photochemical hydrogen evolution with either core-shell CuInS_2_@ZnS QDs or [Ru(bpy)_3_]^2+^ as the sensitizer, ascorbate as the sacrificial donor, and the cobalt complexes **C0–4** as the HECs. Abbreviations: CB = conduction band; VB = valence band; Asc^−^ = ascorbate anion; Asc^●^ = ascorbate radical; RDS = rate-determining step.

Importantly, a characteristic trend in photosynthetic activity has been established using CuInS_2_@ZnS QDs as sensitizers, with complexes **C1** and **C2** bearing electron-withdrawing groups displaying improved light driven HER activity than complexes **C3** and **C4** bearing electron-donating substituents. These results can be explained considering the reduction potential of the CuInS_2_@ZnS QDs and the resulting driving force for the electron transfer to the cobalt complex. To approximately estimate the Gibbs free energy of the catalyst activation step, we employed the potential of the CB in the CuInS_2_@ZnS QDs (E = −1.12 V vs. SCE) [26] and considered for the cobalt complexes the half-wave potential of the catalytic waves in acetonitrile with trifluoroacetic acid as the proton donor (E = −1.03 V for **C0**, −0.80 V for **C1**, −0.98 V for **C2**, −1.11 V for **C3**, and −1.04 V for **C4**) [27] as a reliable measure of the Co(II)/Co(I) reduction in a protic environment (direct measurement of the Co(II)/Co(I) reduction in an aqueous solution is indeed hampered by the rising of the catalytic wave due to proton reduction). The following ∆G values were thus calculated: −0.09 eV for **C0**, −0.32 eV for **C1**, −0.14 eV for **C2**, −0.01 eV for **C3**, and −0.08 eV for **C4**. Interestingly, a superimposable tendency between the driving force and the light-driven hydrogen evolution performance was clearly apparent (*viz*. **C1** > **C2** > **C0** ~ **C4** > **C3**). This evidence was further confirmed by the linear correlation between the logarithm of the QE**_Cn_**/QE**_C0_** ratio at pH 4 (where n = 0–4 depending on the catalyst) and the half-wave potential of the catalytic wave in acetonitrile (Figure 6, red triangles). This parallelism strongly supports that catalyst activation via electron transfer from the CuInS_2_@ZnS QDs represents the rate-determining step of the light-driven hydrogen evolution process in the present three-component system. Consistently, as the driving force for the electron transfer to the catalyst is increased by means of the introduction of electron-withdrawing substituents (*viz.* moving from **C0** to **C2** and eventually to **C1**), catalyst activation becomes more favorable and hydrogen evolution faster. These findings apparently contrast with those obtained using [Ru(bpy)_3_]^2+^ as the sensitizer where the electronic effects exerted by the substituent were not identified as the main source of catalyst diversification within the **C0–4** series. No clear correlation is indeed observed between the logarithm of the QE**_Cn_**/QE**_C0_** ratio and the half-wave potential of the catalytic wave (Figure 6, green triangles). As a matter of fact, in such a three-component system, the large driving force (by more than 0.2 eV) for the catalyst reduction by the photogenerated [Ru(bpy)_3_]^+^ chromophore renders this process sufficiently fast and efficient so that the protonation kinetics become the limiting factor in the resulting light-driven catalysis [27,35]. The proposed mechanistic scenario is depicted in Figure 6.

Hence, the present findings clearly suggest that in photochemical experiments, the electronic effects on molecular catalysts based on transition metal complexes, introduced via chemical functionalization of the ligand periphery, are not general characteristics of the catalytic platform and strongly depend on the experimental conditions in which they are tested. In particular, in the case of photochemical hydrogen evolution by cobalt polypyridine complexes **C0–4** featuring hexadentate ligands, the role of the sensitizer and the reduction ability of the photogenerated reductant appear of remarkable importance in order to establish a specific trend in catalytic performance.

## 4. Materials and Methods

All reagents were purchased from Sigma Aldrich (St. Louis, Missouri; USA) and used as received. Milli-Q water was obtained using a Millipore apparatus, equipped with 0.22 μm filters. The cobalt complexes **C0–4**, available from a previous study [27], were used.

The absorption spectra were recorded by using a Cary 300 UV-Vis (Agilent Technologies, Santa Clara, California, USA) spectrophotometer. The emission spectra were recorded by using an Edinburgh FLS 920 spectrofluorometer. The emission lifetimes were acquired with a custom laser spectrometer comprising a Continuum Surelite II Nd:YAG laser (FWHM 6–8 ns) with a frequency doubling (532 nm) option. Laser excitation was provided at 90° with respect to analysis. The light emitted by the sample was focused onto the entrance slit of a 300 mm focal length Acton SpectraPro 2300i triple grating, flat field, double exit monochromator equipped with a photomultiplier detector (Hamamatsu R3896). The signals from the photomultiplier (kinetic traces) were processed by means of a TeledyneLeCroy 604Zi (400 MHz, 20 GS·s^−1^) digital oscilloscope. The high-resolution transmission electron microscope (HR-TEM) characterization was carried out by using an FEI Tecnai F20 field emission instrument, operated at 200 keV. Energy dispersive X-ray spectroscopy (EDS) was employed to detect sample composition. TEM grids were prepared by drop casting the QD solution onto a Ni grid coated with a carbon film after evaporation of the solvent at 100 °C for 10 min. The light-driven hydrogen evolution experiments were carried out upon continuous visible-light irradiation of a reactor containing the solution (a 10 mm pathlength pyrex glass cuvette with headspace obtained from a round-bottom flask). Solutions were purged using argon gas for 15 min prior to irradiation. A 175 W Xenon light (CERMAX) was used as the light source, a cut-off filter at λ = 400 nm and a hot mirror were employed to provide suitable irradiation between 400 and 700 nm. The gas phase of the reaction vessel was analyzed by using an Agilent Technologies 490 microGC equipped with a 5 Å molecular sieve column (10 m), a thermal conductivity detector, and by using argon as the carrier gas. Additional details of the setup and procedures used for the hydrogen evolution experiments can be found in previous reports [40,41,42].

**Synthesis of CuInS_2_@ZnS QDs**. The synthesis of the QDs was taken from the literature [26]; 20 mL of water were degassed using nitrogen, and 307 mg glutathione and 12 mg Cu(NO_3_)_2_·3H_2_O were then added to yield solution A. In a separate vessel, 30 mL water was degassed using nitrogen and 60 mg In (NO_3_)_3_·5H_2_O were added to yield solution B. Solutions A and B were then mixed under nitrogen and the pH corrected to about 9–10 using 1 M NaOH until a transparent solution was achieved. 2 mL of a 0.2 M Na_2_S·9H_2_O solution were then added and the resulting mixture was heated by microwave according to the following ramp procedure: (a) RT to 100 °C (1 min); (b) 100 °C (6 min), (c) RT (20 min). After cooling down to RT, 2 mL of a 0.2 M Na_2_S·9H_2_O solution and 2 mL of a 0.2 M (CH_3_COO)_2_Zn·9H_2_O solution were added, and the resulting solution was heated by microwave following the same ramp procedure. After cooling down, the CuInS_2_@ZnS QDs were obtained by precipitation with acetone, redissolution in water (pH 9), and final reprecipitation with acetone. The obtained solid sample was dissolved in a small amount of water and stored at 4 °C in a cooler.

## 5. Conclusions

The series of cobalt complexes **C0–4** featuring differently-substituted hexadentate ligands have been examined as catalysts for light-driven hydrogen evolution in an aqueous solution in combination with core-shell CuInS_2_@ZnS QDs as sensitizers and ascorbate as the sacrificial electron donor. Sustained hydrogen production has been recorded for all the molecular catalysts tested under the present experimental conditions, with complex **C1** featuring electron-withdrawing –CF_3_ substituents on the bipyridines leading to the highest performances (maximum TON = 329, TOF = 53 h^−1^, QE = 0.29%) within the series. A specific tendency in photosynthetic activity has been observed which parallels the required driving force for the catalyst activation step. This evidence contrasts previously obtained results with the same class of catalysts using the standard [Ru(bpy)_3_]^2+^/ascorbate photochemical system and has been explained considering diverse rate-determining steps in the photochemical reaction for the two different three-component systems, mainly associated with the different reduction potentials of the photogenerated reducing agent. The present results thus suggest that electronic effects in light-driven molecular catalysis are not exclusive features of the catalytic platform and must take into account the entire photochemical system as well as the experimental conditions. Tentative approaches aimed at generalizing these observations are currently planned in our labs.

## Data Availability

Not applicable.

## Supplementary Materials

The following supporting information can be downloaded at: https://www.mdpi.com/article/10.3390/molecules27238277/s1, Figure S1: High-resolution TEM image of aqueous CuInS2@ZnS QDs drop casted onto a Ni grid followed by drying at 100 °C; Figure S2. High-resolution TEM image of aqueous CuInS2@ZnS QDs drop casted onto a Ni grid followed by drying at 100 °C after a few minutes’ beam irradiation and diffraction pattern obtained in one of the highlighted regions (inset); Figure S3. EDS spectrum of aqueous CuInS2@ZnS QDs drop casted onto a Ni grid followed by drying at 100 °C; Figure S4. Luminescence decays at (a) 650 nm, (b) 700 nm, and (c) 740 nm obtained by laser flash photolysis (excitation at 532 nm) of aqueous CuInS2@ZnS QDs; Figure S5. Luminescence of aqueous CuInS2@ZnS QDs and fitting using two Gaussian functions; Figure S6. Kinetics of hydrogen evolution obtained upon visible light irradiation of aqueous solutions (5 mL) containing 0.11 mM CuInS2@ZnS QDs, 0.5 M ascorbate, 40 µM **C0** at different pH; Figure S7. I0/I ratio vs. catalyst concentration for the quenching of CuInS2@ZnS QDs luminescence by complex **C1**; Figure S8. I0/I ratio vs. catalyst concentration for the quenching of CuInS2@ZnS QDs luminescence by complex **C2**; Figure S9. I0/I ratio vs. catalyst concentration for the quenching of CuInS2@ZnS QDs luminescence by complex **C3**; Figure S10. I0/I ratio vs. catalyst concentration for the quenching of CuInS2@ZnS QDs luminescence by complex **C4**; Figure S11. I0/I ratio vs. concentration for the quenching of CuInS2@ZnS QDs luminescence by the ascorbate donor.
