# Supplementary material for: Rethinking Electronic Effects in Photochemical Hydrogen Evolution Using CuInS2@ZnS Quantum Dots Sensitizers
Source: Molecules. 2022 Nov 27;27(23):8277. doi: 10.3390/molecules27238277 (PMC9735784; doi:10.3390/molecules27238277)
Supplement: Supplementary file 1 [file molecules-27-08277-s001.zip › molecules-2029513-supplementary.pdf]

Supplementary Material

Rethinking electronic effects in photochemical hydrogen evolution using $CuInS_2$@ZnS quantum dots sensitizers

Antonio Orlando,[1,2,3] *Fiorella Lucarini,*[4,5] *Elisabetta Benazzi,*[1,6] *Federico Droghetti,*[1] *Albert Ruggi,*[4,*] *Mirco Natali*[1,*]

[1] Department of Chemical, Pharmaceutical and Agricultural Sciences (DOCPAS), University of Ferrara, Via L. Borsari 46, 44121 Ferrara, Italy.

[2] Current address: Faculty of Science and Technology, Free University of Bolzano-Bozen, Piazza Università 5, 39100 Bolzano, Italy.

[3] Current address: MNF Unit, Sensors and Devices Center, Bruno Kessler Foundation, Via Sommarive 18, 38123 Trento, Italy.

[4] Department of Chemistry, University of Fribourg, Chemin de Musée 9, CH-1700 Fribourg, Switzerland.

[5] Current address: HEIA-FR, Bd. De Pérolles 80, CH-1700 Fribourg, Switzerland.

[6] Current address: California Institute of Technology, 1200 East California Blvd., 91125 Pasadena, CA, U.S.A.

* Correspondence: A. R., albert.ruggi@unifr.ch; M. N., mirco.natali@unife.it

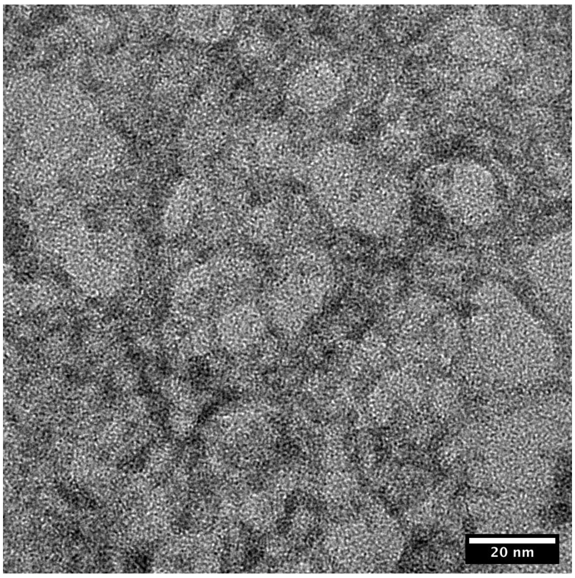

Figure S1. High-resolution TEM image of aqueous $CuInS_2$@ZnS QDs drop casted onto a Ni grid followed by drying at 100°C.

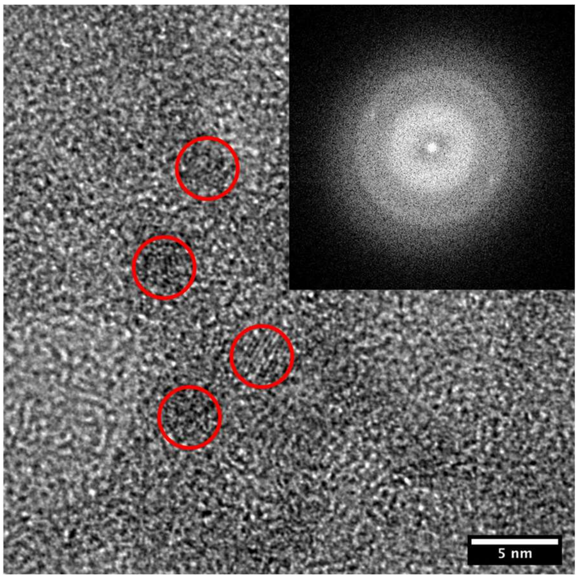

Figure S2. High-resolution TEM image of aqueous $CuInS_2$@ZnS QDs drop casted onto a Ni grid followed by drying at 100°C after a few minutes' beam irradiation and diffraction pattern obtained in one of the highlighted regions (inset).

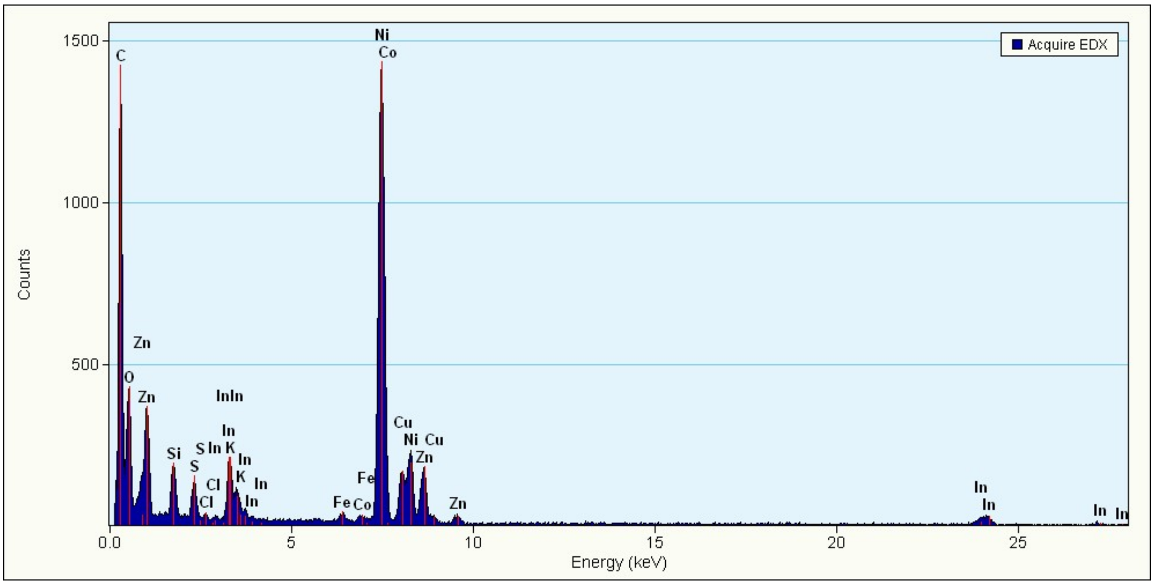

Figure S3. EDS spectrum of aqueous $CuInS_2$@ZnS QDs drop casted onto a Ni grid followed by drying at 100°C.

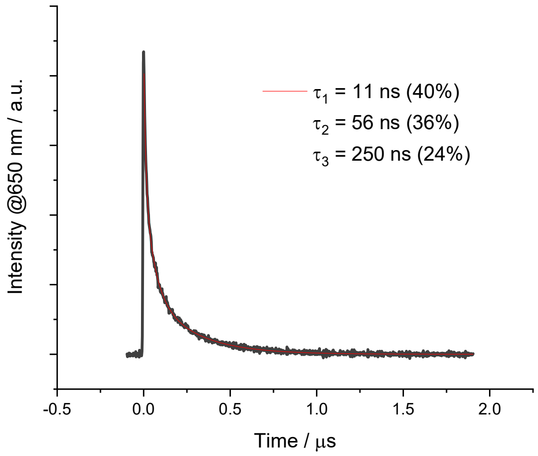

a)

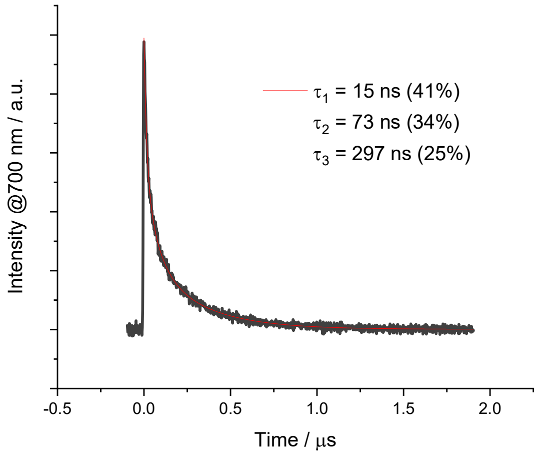

b)

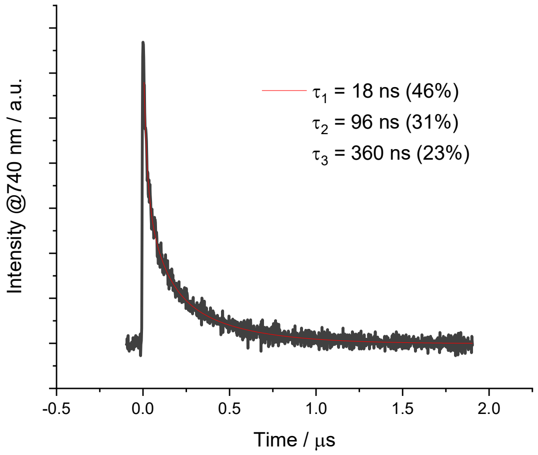

c)

Figure S4. Luminescence decays at a) 650 nm, b) 700 nm, and c) 740 nm obtained by laser flash photolysis (excitation at 532 nm) of aqueous $CuInS_2$@ZnS QDs.

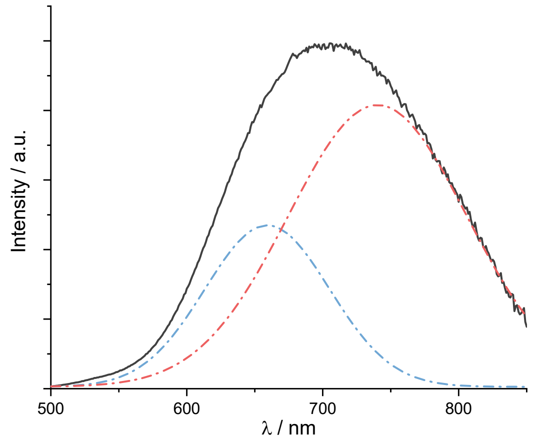

Figure S5. Luminescence of aqueous $CuInS_2$@ZnS QDs and fitting using two Gaussian functions.

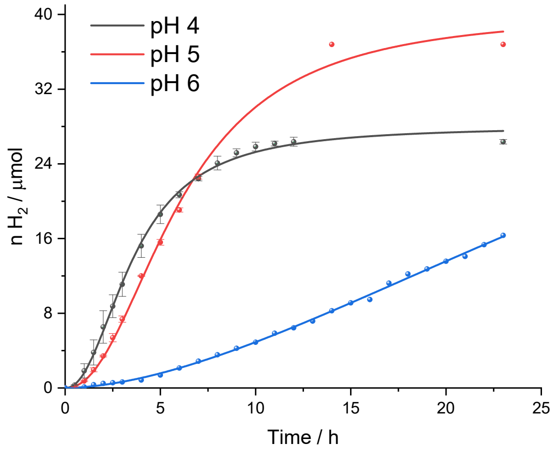

Figure S6. Kinetics of hydrogen evolution obtained upon visible light irradiation of aqueous solutions (5 mL) containing 0.11 mM $CuInS_2$@ZnS QDs, 0.5 M ascorbate, 40 μM **C0** at different pH.

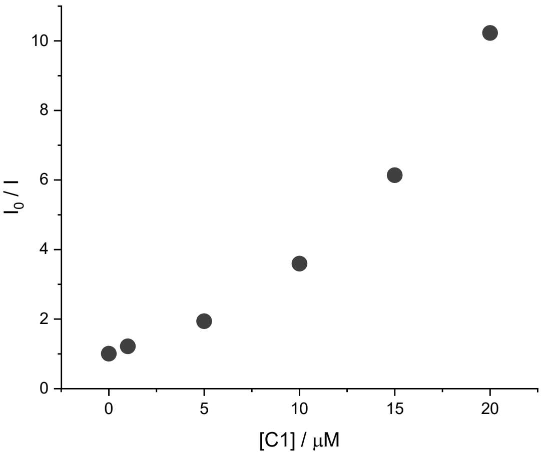

Figure S7. I_0 / I ratio vs. catalyst concentration for the quenching of $CuInS_2$@ZnS QDs luminescence by complex **C1**.

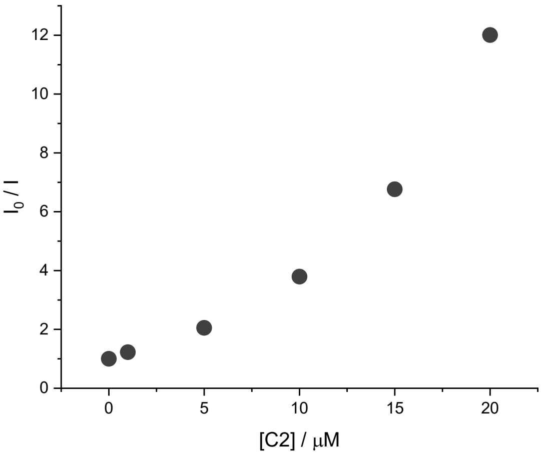

Figure S8. I_0 / I ratio vs. catalyst concentration for the quenching of $CuInS_2$@ZnS QDs luminescence by complex **C2**.

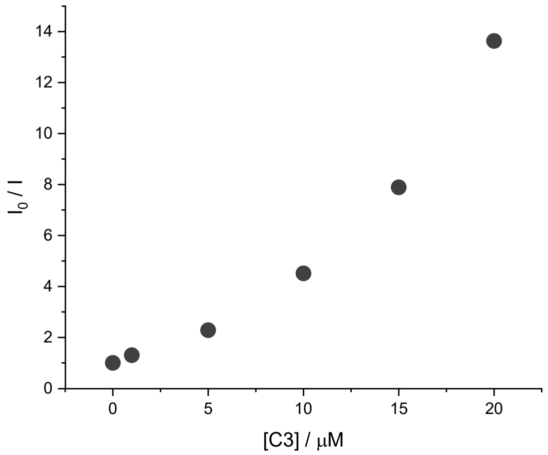

Figure S9. I_0 / I ratio vs. catalyst concentration for the quenching of $CuInS_2$@ZnS QDs luminescence by complex **C3**.

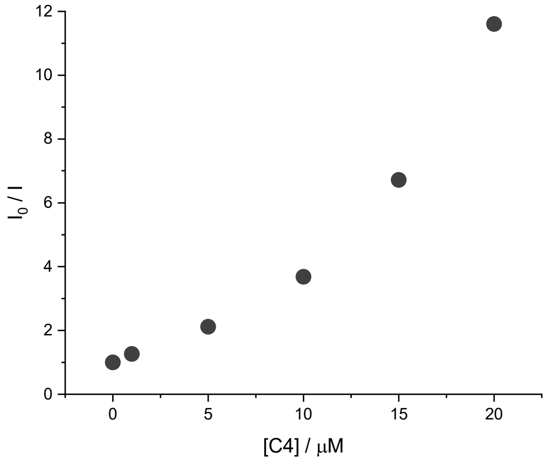

Figure S10. I_0 / I ratio vs. catalyst concentration for the quenching of $CuInS_2$@ZnS QDs luminescence by complex **C4**.

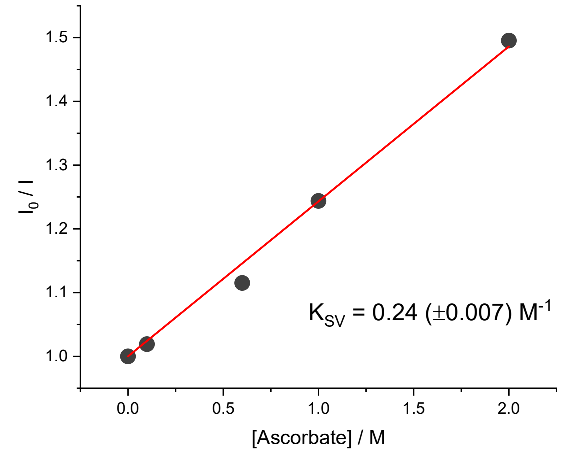

Figure S11. I_0 / I ratio vs. concentration for the quenching of $CuInS_2$@ZnS QDs luminescence by the ascorbate donor.
